# Dielectric Screening inside Carbon Nanotubes

**DOI:** 10.1021/acs.nanolett.4c01668

**Published:** 2024-06-24

**Authors:** Georgy Gordeev, Sören Wasserroth, Han Li, Ado Jorio, Benjamin S. Flavel, Stephanie Reich

**Affiliations:** † Department of Physics, Freie Universität Berlin, Arnimallee 14, 14195 Berlin, Germany; ‡ Department of Physics and Materials Science, University of Luxembourg, Rue du Brill 41, L-4422 Belvaux, Luxembourg; ¶ Institute of Nanotechnology, Karlsruhe Institute of Technology, Hermann-von-Helmholtz-Platz 1, 76344 Eggenstein-Leopoldshafen, Germany; § Department of Mechanical and Materials Engineering, University of Turku, Vesilinnantie 5, 20500 Turku, Finland; ∥ Departamento de Física, Universidade Federal de Minas Gerais, Belo Horizonte, Minas Gerais 30123-970, Brazil; ⊥ Turku Collegium for Science, Medicine and Technology, University of Turku, FI-20520 Turku, Finland

**Keywords:** dielectric screening, excitons, one-dimensional heterostructures, double-walled nanotubes, resonant Raman, carbon nanotubes

## Abstract

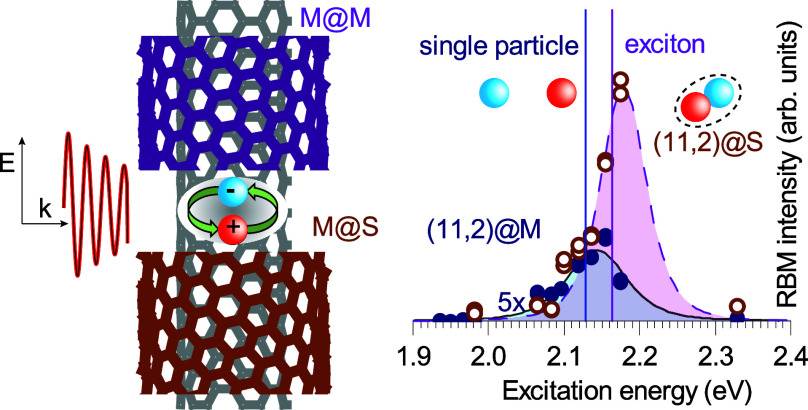

Dielectric screening plays a vital role in determining physical properties at the nanoscale and affects our ability to detect and characterize nanomaterials using optical techniques. We study how dielectric screening changes electromagnetic fields and many-body effects in nanostructures encapsulated inside carbon nanotubes. First, we show that metallic outer walls reduce the scattering intensity of the inner tube by 2 orders of magnitude compared to that of air-suspended inner tubes, in line with our local field calculations. Second, we find that the dielectric shift of the optical transition energies in the inner walls is greater when the outer tube is metallic than when it is semiconducting. The magnitude of the shift suggests that the excitons in small-diameter inner metallic tubes are thermally dissociated at room temperature if the outer tube is also metallic, and in essence, we observe band-to-band transitions in thin metallic double-walled nanotubes.

Carbon nanotubes (CNTs) can serve as a one-atom thick container for molecules and one-dimensional (1D) crystals.^1−8^ A CNT container may be exploited as a drug carrier and local sensor.^9,10^ It also provides a unique environment for tailoring and studying encapsulated materials.^11^ For example, water changes its dielectric behavior and viscosity inside tubes and can adopt new phases.^12,13^ Such water@CNT channels were used for ion transport to potentially model biological systems like transmembrane proteins.^14^ In addition, CNTs act as templates to order and align molecules, which lead to micrometer-sized single-file *J* aggregates or molecular chains with huge optical nonlinearities.^3,15^ Despite extensive studies of filled nanotubes and hybrid 1D systems, the environment produced by an encapsulating CNT remains mysterious. For instance, 1D chains of dye molecules or carbon atoms inside a nanotube yield record-high Raman cross sections.^3,16^ This enhancement may be due to intrinsic effects, molecule–molecule interaction, molecule–wall coupling such as state hybridization, or dielectric effects by the nanotube wall. In the case of 1D molecular crystals and carbon chains, it is impossible to discriminate between the different effects, because they exist only inside the CNTs and cannot be extracted and studied under ambient conditions. There are some indications of systematic changes in materials inside CNTs. For example, the nanotube walls affect the electromagnetic (EM) field inside the CNT. C_60_@CNT demonstrated different depolarization ratios,^17^ but whether this was related to the depolarization of the EM field or strain remained unclear. On the contrary, single-walled nanotubes are stable under ambient conditions or can be an inner part of a double-walled CNT (DWCNT).^18^ DWCNTs can serve as ideal probes of the environment produced by a nanotube, because an inner wall nanotube may be easily referenced to an SWCNT^19^ and will probe the environment produced by the outer wall. The nanotube walls can alter the many-body effects of encapsulated materials, because electron–hole interactions are subjected to exterior screening.^20^ This may fundamentally change how collective states form, for example, for 1D *J* aggregates inside tubes. It has often been suggested that nanotube excitons are tuned by interior filling,^4,21^ but the inverse effect, in which a CNT affects the excitons of encapsulated species has not received much attention. For example, the excitonic series of carbyne are screened by the CNT wall,^22^ but the intrinsic exciton energies remain unknown. This effect may become particularly noteworthy for metallic outer CNTs, because the metallic species are expected to provide a much denser dielectric environment^23,24^ and the electrons in the metallic wall screen the charge in the opposite one.^25^ Extreme screening by an outer tube was predicted to reduce the excitons of the inner tube to single-particle excitation in small-diameter CNTs,^26,27^ but experimental evidence has been lacking until now.

In this work, we study dielectric screening by metallic CNTs using resonant Raman scattering on DWCNTs. The EM screening by the outer wall reduces the inner tube Raman intensity in metallic@metallic DWCNTs by a factor of ≲100, in agreement with a dielectric model of a hollow cylinder in the quasistatic approximation and a dielectric constant ε_oT_ of 9–10 for the outer metallic wall. In addition, we compare the inner tube excitonic transition energies for semiconducting and metallic hosts. The transition energies shift to lower energies compared to those of SWCNTs, which we analyze in the framework of the dielectric screening model. The magnitude of the shift in the transition energies of small inner metallic tubes is compatible with a complete dissociation of the exciton due to dielectric screening.

We consider a situation in which a single-walled carbon nanotube is embedded in another tube (Figure 1a). Neglecting direct tube–tube coupling, the outer tube creates a dielectric environment that changes the optical response of the inner tube in two ways. The outer tube reduces the amplitude of an external EM field for the inner wall and the field orientation so that it is predominantly polarized along the axis.^20^ The change in polarization direction arises from what has been coined the antenna effect,^20^ i.e., the fact that a nanoscale cylinder screens an EM field that is polarized perpendicular to its axis. The change in the electric field amplitude is determined by the effective dielectric constant due to the presence of the outer wall, as shown in Figure 1a. Local field *E*_loc_ inside the outer tube can be derived from classical electrodynamics. We model the outer tube as a hollow cylinder with diameter *d*, wall thickness *t* (∼0.32 nm), and dielectric constant ε_oT_ (see Figure 1a). To calculate the contribution to ε, we requite not the mechanical thickness^28^ but rather a dielectric thickness of the wall. The wavelength of light (530–850 nm) is much larger than the CNT diameter (2 nm), which indicates the quasistatic regime and the field inside the cylinder can be approximated as^29^
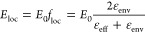
1where *f*_loc_ is the local field factor, *E*_0_ is the external electric field, and ε_env_ is a dielectric constant of the environment around the outer tube. Effective dielectric constant ε_eff_ arises from the combined dielectric effect of the outer wall itself with ε_oT_ and inner core ε_i_ (ε_i_ = 1 for an empty tube). We calculate it according to Maxwell–Garnet mixing^30−32^
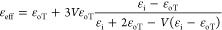
2where  is the volume fraction of the empty part of the cylinder. For a CNT with a *d* of 2 nm, we find *V* = 0.54. Assuming ε_i_ = 1 and ε_oT_ = 10, we obtain the effective dielectric constant ε_eff_ ≈ 5.

**Figure 1:**
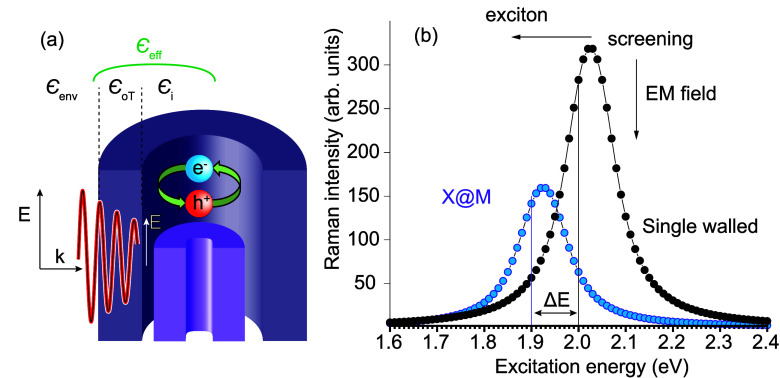
Dielectric screening in DWCNTs. (a) Model of a DWCNT as a cylinder of two dielectric walls. The EM field at the position of the inner tube is modulated by the outer wall dielectric constant. (b) The dielectric effect manifests in the resonant Raman profiles of the (*n*_0_, *m*_0_) inner wall (blue) compared to corresponding SWCNT (black) computed by eq 4. The profile of DWCNTs is red-shifted due to exciton screening, and the amplitude is reduced by the local field factor compared to that of the SWCNT.

Effective dielectric constant ε_eff_ changes the optical transition energy as given by^33,34^
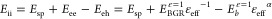
3

Single-particle band gap *E*_sp_ is independent of that of ε_eff_. *E*_BGR_ is the electron–electron interaction energy for the unscreened system.^34^ Electron–electron correlation *E*_ee_ scales as ε_eff_^–1^ for small electron wave vectors according to the Coulomb potential.^35^ The electron–hole *E*_eh_ interaction is more complex and leads back to a hydrogen atom problem in one dimension, where a cutoff potential of ∼1/|*z*_0_ + *z*| is typically introduced to converge the ground state.^36^ This yields the equation *E*_eh_ = *R*_h_^*^/λ^2^, where effective Rydberg radius *R*_h_^*^ depends on ε_eff_ and λ varies with potential cutoff *z*_0_ as a function of ε_eff_. When these two factors are combined, one obtains *E*_eh_ ∼ ε_eff_^α^, where α = 1.2–1.4 is a semiempirical scaling factor.^34,37,38^ The different scaling of the electron–electron and electron–hole interactions yields an overall shift of the optical excitation energy as given by eq 3.

The optical response of the inner tube is sensitive to the change in the local field and the exciton transition energies caused by the outer tube. In principle, the optical effects can be studied by any optical techniques such as photoluminescence excitation,^39^ direct absorption,^40,41^ or resonant Raman spectroscopy.^42−44^ However, photoluminescence is present in only semiconducting inner tubes; in addition, it is strongly quenched by the outer wall. Optical absorption is challenging to measure experimentally due to the small cross sections and signal overlap in chiral mixtures. In contrast, resonant Raman scattering provides a sufficient signal for metallic and semiconducting walls up to the single-tube level.^45^ Resonant Raman spectroscopy of the radial breathing modes (RBMs) is a key method for following the optical and vibrational changes in samples containing mixed and single chiralities.^20,42−44^ The phonon energy of the RBM depends on tube diameter^46^, allowing one to distinguish between nanotubes of different sizes (e.g., inner and outer tube).^47,48^ A van der Waals parameter *c*_a_ depends on the filling, exterior functionalization, and wall-to-wall interactions.^4,18,43,49,50^

The optical transitions of inner walls and EM screening effects are probed via resonant Raman spectroscopy, i.e., the dependence of the scattering intensity on laser excitation.^20,42,43,49^ The screening by the outer tube manifests in a shift of the resonant Raman profile that is determined by optical transition energy *E*_ii_, as shown in Figure 1b. The change in the EM field intensity reaching the inner tubes reduces the amplitude of the resonant Raman profile varying with laser excitation energy *E*_l_ as
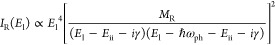
4where *M*_R_ is the combined Raman matrix element. It is given by the equation *M*_R_ = *M*_ex–pt_^2^*M*_ex–RBM_ as the product of exciton–photon *M*_ex–pt_ and exciton–phonon matrix elements *M*_ex–RBM_.^51^*M*_ex–pt(in)_ depends linearly on the local electric field amplitude and thus scales with *f*_loc_, which leads to the relationship *I*_R_ ∝ *f*_loc_^4^. Broadening factor γ is inversely proportional to the exciton lifetime. Overall, higher screening is expected to reduce the Raman intensity and produce a red-shift of the resonance, as shown in Figure 1b.

Figure 2a shows the experimental RBMs of M@M and M@S DWCNT samples (inner@outer); i.e., all inner tubes are metallic, and the outer tubes are metallic in the M@M sample but semiconducting in the M@S sample. We confirm the semiconducting or metallic character of the DWCNT wall from the RBM spectra. We first divide the frequencies into a range of outer tubes (*ℏω*_RBM_ < 200 cm^–1^) and inner tubes (*ℏω*_RBM_ > 200 cm^–1^). We deduce the metallic and semiconducting character of the inner tube from the excitation energy dependence and frequency, because only resonant tubes show measurable intensity.^20,42,43^ Thereby, we identify the RBM frequency ranges for metallic (labeled blue) and semiconducting (orange) species (Figure 2a). Individual RBMs are grouped by 2*n* + *m* laola families, as shown in panels b and c of Figure 2.^42^

**Figure 2:**
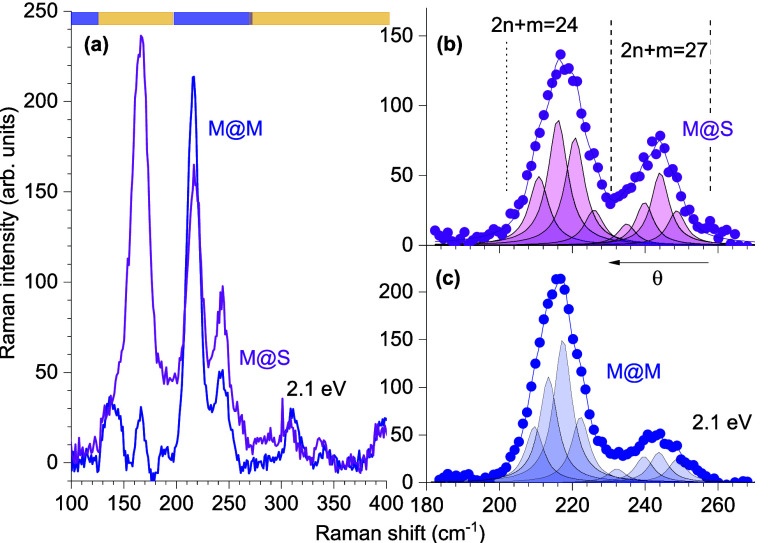
Radial breathing modes in M@M (blue) and M@S (purple) nanotubes excited with a 2.1 eV laser. (a) RBM spectra in the region between 100 and 400 cm^–1^. The top line roughly divides expected RBMs from metallic (blue) and semiconducting (orange) walls. Inner wall RBM fitting in the (b) M@S sample and (c) M@M sample. The vertical lines divide different 2*n* + *m* laola families. The arrow indicates the increase in the chiral angle within a laola group.

The RBM frequencies of the DWCNTs with metallic and mixed walls were only slightly shifted (a few inverse centimeters or less) compared to the corresponding SWCNT, and no splitting, which is characteristic for S@S, was observed. This can be explained by the electronic states of the inner and outer walls in M@M and M@S being energetically well separated, which reduces the level of moiré coupling.^18^ We fitted the diameter dependence of the RBM frequencies and obtained higher van der Walls constants *c*_a_^M@M^ = 6.6 × 10^–2^  nm^–2^ and *c*_a_^M@S^ = 7.5 × 10^–2^ nm^–2^ compared to *c*_a_^SWCNTs^ = 5.7 × 10^–2^ nm^–2^.^46^ The RBM frequency shifts of the inner metallic tubes result from a changed intercept of the RBM diameter dependence, which is associated with environmental effects, as a change in solvent or nanotube bundling.^42,52^

The EM screening effect by the outer tubes is manifested in the measured RBM intensities from the inner walls. We first selected spectra with the highest RBM intensities for the inner and outer species in S@S, M@S, and M@M samples (Figure 3a–c) [the S@M sample had a lower sorting purity and will not be considered here (see Experimental Methods in the Supporting Information)]. When the outer tubes are semiconducting, the maximum RBM intensity of the inner tube is comparable to or even stronger than that of the outer species (Figure 3a,b). This is expected, because the RBM intensities scale with the inverse of the nanotube diameter.^19,20,53^ In sharp contrast, the integrated intensity of the inner tube is 1 order of magnitude smaller with a metallic outer tube in the M@M sample (Figure 3c). To study the intensities in detail, we measured the full resonant profiles of the inner and outer walls. Figure 3d compares the exemplary Raman profiles of the (12,0) inner tube (ℏω_RBM_^i^ = 249 cm^–1^, and *E*_11_ = 2.08 eV) and the (12,12) outer tube (ℏω_RBM_^oT^ = 144 cm^–1^, and *E*_11_ = 1.48 eV). The difference between the Raman intensities is a striking factor of 30 (Figure 3d). Similar trends were observed for the other M@M walls as shown in Figure 3e, where Raman intensity *I*_R_ is plotted versus the wall diameter; the inner wall intensities in the range 0.8–1.8 nm are ≤100 times smaller.

**Figure 3:**
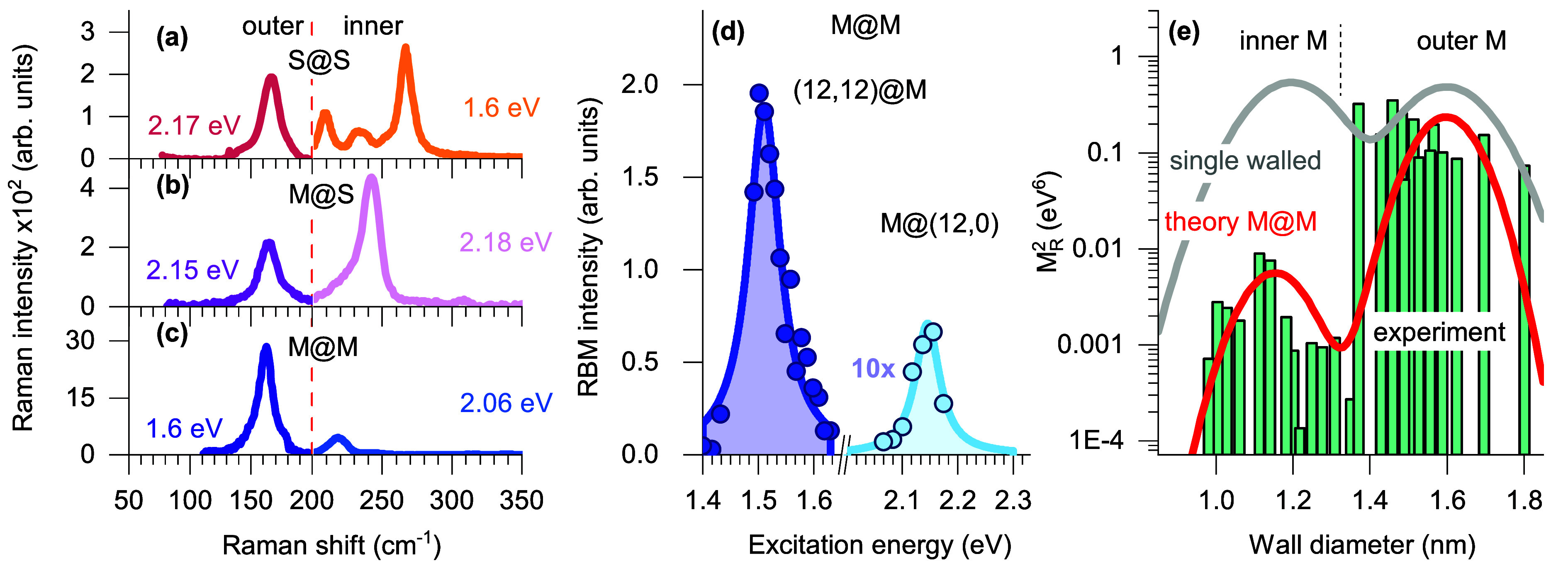
Electromagnetic field screening in the M@M sample. (a–c) Relative intensities of the RBMs originating from the inner and outer walls in S@S, M@S, and, M@M samples, respectively. The inner and outer walls excited at different energies are separated by a vertical line at 200 cm^–1^. (d) Resonant Raman profile of the inner (12,0)@M and outer M@(12,12) wall, with RBMs at 249 and 144 cm^–1^, respectively. Symbols are experimental data, and lines are fits by eq 4. (e) Green bars: measured Raman intensities (*M*_R_^2^) as a function of wall diameter, estimated from resonant Raman profiles (note the logarithmic scale of the *y* axis). Red line: calculated Raman intensity ε_oT_ = 10 and ε_oT_ = 1, by eq 1. Gray line: intrinsic *M*_R_^2^ without the correction factor.

The experimentally observed variation of Raman intensity is reproduced in eq 1 with dielectric constants ε_oT_ = 9–10 and ε_in_ = 1. The RBM intensity is plotted in Figure 3e with and without local field factor correction (details in the Supporting Information). As an alternative to dielectric screening, strong doping can change the Raman intensities,^54,55^ yet the LO phonon line width is relatively high at 72–73 cm^–1^, which indicates moderate doping of 40 meV in the M@M and M@S samples^56−58^ (see the Supporting Information). Overall, we see good agreement between theory and experiment in terms of intensities, whereas the dependence of *M*_R_^2^ without local field correction (gray line in Figure 3e) disagrees with experiment. The experimental dielectric constant ε_oT_ of 10 compares reasonably well with the theoretical ε_oT_^th^ value of 16^23^ and previous experimental reports where it was deduced from the transition energy shift in a dielectric environment. Araujo et al.^59^ reported an ε_eff_ of 7.7^59,60^ for a *d* of 1 nm, and we obtain an ε_eff_ of 7 in our model.

The EM screening of metallic CNTs can hide Raman and nonlinear signals originating from the inner walls. *d* values of 1–2 nm and an ε_oT_ of 10 reduce the amplitude of the electric field within the tube by *f*_loc_ ≈ 0.36, which leads to *f*_loc_^4^ ≈ 0.017. Thus, Raman signals are reduced by 2 orders of magnitude, which may be the reason why carbyne chains so far have been observed in only individual semiconducting DWCNTs.^61,62^ This also means that the semiconducting container will be favored during in situ Raman spectroscopy monitoring of chemical reactions, e.g., in the fusion of molecules to graphene ribbons and inner tubes.^63,64^ Finally, when the laser energy matches the optical transition of the outer wall, ε_eff_ will trigger an active dielectric screening.

Now we turn to the analysis of the exciton screening effects and determine the transition energies of inner CNTs from the resonant Raman profiles. We start with the RBM at 244 cm^–1^ in Figure 2b, belonging to (11,2)@S. The integrated area of the (11,2)@S RBM is plotted as a function of excitation energy in Figure 4b. The intensity increases when the laser approaches the *E*_11(L)_ transition energy of the nanotube. We quantify this energy by fitting the (11,2)@S Raman profile by eq 4 and find a transition energy *E*_11(L)_ of 2.16 eV, marked by a vertical red line. The transition energy in the DWCNTs is comparable to that in the (11,2) SWCNTs (2.12 eV). In single-walled CNTs, *E*_11(L)_ may depend on many factors, surfactant type and filling, compared to that of DWCNTs, in which the exclusive environment is the outer wall that can be either semiconducting or metallic.

**Figure 4:**
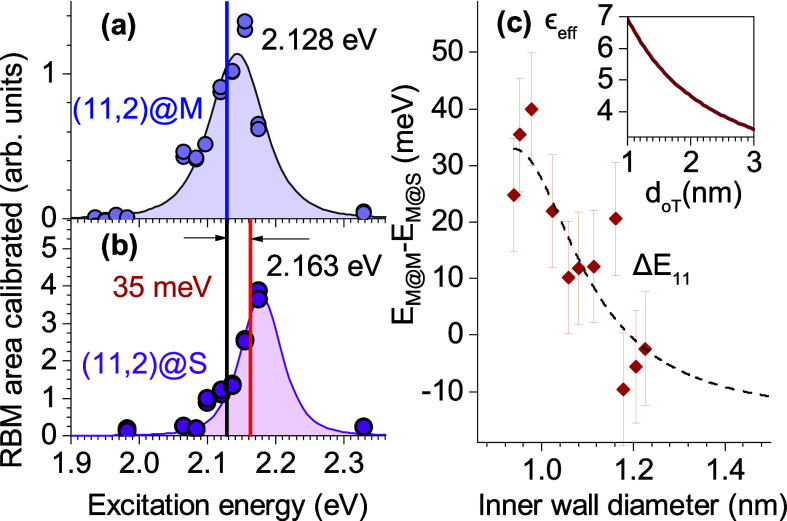
Exciton screening in the S@M and M@M samples studied by resonant Raman scattering. Resonant Raman profiles of (a) (11,2)@M and (b) (11,2)@S. Symbols represent experimental data, and lines fits by eq 4. The positions of the transition energies are marked by vertical lines. (c) Transition energy shifts measured in the identical inner tubes in the M@M and M@S samples (listed in Table 1). The inset shows diameter dependence ε_eff_ vs the outer wall diameter for ε_oT_ and ε_i_ values of 10 and 1, respectively.

The metallic outer wall induces a larger shift in transition energy of the inner wall exciton compared to a semiconducting outer wall. The resonant Raman profiles for (11,2)@M and (11,2)@S are plotted in Figure 4a,b, respectively. In the (11,2)@M sample, we find *E*_11(L)_^(11,2)@M^ = 2.163 eV, which is smaller by 35 meV than *E*_11(L)_^(11,2)@S^. Because the Raman frequencies of the inner walls are identical, both at ∼244 cm^–1^, moiré coupling can be neglected^18^ and the dominant origin of the energy shift must be dielectric screening, as expected from eq 3. Lifetime broadening γ of the Raman profile for (11,2)@M is 69 meV, which compares well with our previous observation in the S@S sample (γ_S@S_ = 60 meV).^18^ The broadening is smaller that expected in bundles of metallic SWCNTs, typically between 90 and 100 meV;^52,58^ therefore, we estimate the amount of single-walled impurities arising from the sonication process^65,66^ to be small and confirm that profiles in panels a and c of Figure 4 belong to DWCNTs (details in the Supporting Information).

The red-shift in the transition energy is stronger for small-diameter inner nanotubes. We plot the shift of the transition energy between the M@S and M@M samples in Figure 4c. The magnitude of the shift decreases from ∼40 meV for a *d* of 1 nm to nearly zero for a *d* of 1.2 nm. The shift scales with effective dielectric constant ε_eff_, plotted as a function of inner wall diameter for ε_oT_ and ε_i_ values of 10 and 1, respectively, in the inset of Figure 4c. The shift direction compares well with previous theoretical calculations^27^ and experiments in large-diameter S walls,^67^ as listed in Table 1. The scattering of the symbols on the order of 10 meV is mainly due to the weak moiré effects, which also induce a slight negative shift in some cases.

**Table 1:** Summary of RBM Frequencies *ℏω*_ix_ and Transition Energies *E*_11(L)_^ix^ Extracted from Fitting Resonant Raman Profiles, Where ix = SW (single-walled), M@M, or M@S [Δ*E*_11(L)_^M@X^ = *E*_11_^M@S^ – *E*_11_^M@M^]

2*n* + *m*	(*n*,*m*)	*ℏω*_SW_ (cm^–1^)	*ℏω*_M@M_ (cm^–1^)	*ℏω*_M@S_ (cm^–1^)	*E*_11(L)_^SW^ (eV)	*E*_11(L)_^M@M^ (eV)	*E*_11(L)_^M@S^ (eV)	Δ*E*_11(L)_^M@X^ (meV)
24	(9,6)	228	232.0	231.7	2.25	2.134	2.156	22
	(10,4)	238	238.8	238.9	2.24	2.132	2.172	40
	(11,2)	244	244.2	244.5	2.22	2.128	2.163	35
	(12,0)	247	249.4	249.6	2.21	2.128	2.152	25
27	(10,7)	203	207	210	2.04	2.050	2.070	21
	(11,5)	211	210	213	2.08	2.050	2.062	12
	(12,3)	217	217	217.9	2.07	2.037	2.049	12
	(13,1)	221	222	222.4	2.07	2.024	2.034	10
30	(13,4)	193.5	195.9	197.6	1.93	1.902	1.899	–2
	(14,2)	196.3	199.8	201.9	1.92	1.901	1.896	–6
	(15,0)	200.4	204.4	206.0	1.88	1.904	1.894	–10

Excitons in thinner M@M inner walls can be reduced to single particles by extreme outer wall screening. The thermal dissociation of the excitons occurs when the exciton binding energy becomes comparable to the thermal energy at room temperature *k*_B_*T*_293_ = 25 meV. With an ε_oT_ of 10, we obtain an ε_eff_ of 4.5 and a reduction in binding energy from 114 to 15 meV. This yields an *E*_11(L)_ red-shift of 19 meV, given by eq 3. These or greater red-shifts are indeed observed for inner CNTs with *d* values of <1.03 nm in our sample (Figure 3 and Table 1), indicating that we observe single-particle band gaps in the inner M@M walls. A more efficient screening of the inner wall exciton by the outer metallic wall than by the semiconducting wall was theoretically studied in ref (27). We find similar effects; in addition, when both walls are metallic, the screening yields exciton dissociation at room temperature.

Many fundamental properties of CNTs are governed by excitons, including absorption,^23^ emission,^68^ and Raman scattering.^44,69^ It would be extremely interesting to perform such experiments on screened excitons, which are available in the inner walls of M@M DWCNTs. For example, we expect asymmetric absorption peaks, identical energies in one- and two-photon luminescence excitation spectroscopy, and a change in the relative intensity of the incoming and outgoing G mode Raman resonances.^26,44,68,69^ The fragility of the excitonic states in partly or fully metallic DWCNTs makes them unlikely candidates for preparing exciton condensates and exciton insulators, where a better choice would be fully semiconducting species with strong moiré effects.^18^

The dielectric effects may be used to control materials encapsulated inside carbon nanotubes via screening. The optical transition energies of the molecules and carbon chains are ruled by excitonic effects.^70^ Such effects would manifest in an optical energy shift when confined inside metallic nanotubes compared with semiconducting ones. For example, in linear carbon chains, we would expect a deviation from the linear behavior between the Raman mode frequency and the transition energy.^71^ To date, individual single-carbon chains have been reported in only semiconducting CNT containers.^16,72^ This is likely related to their localization method, where first the lateral Raman maps are analyzed for the strongest Raman signal. As we showed, the Raman signals inside the metallic shells are much smaller; therefore, improved localization methods are required to target excitonic effects in 1D chains.

In conclusion, dielectric screening plays an important role in DWCNT; its effects are 2-fold as it modulates many-body effects and alternates the electric field inside the outer tube. Many-body effects manifest in the energetic positions of the excitons. We measured inner wall exciton energies by resonant Raman spectroscopy for semiconducting and metallic outer walls. In metallic outer walls, we found an additional red-shift of ≤40 meV, compared to that in semiconducting outer walls. The optical resonances of inner metallic walls most likely originate from band-to-band excitations because the excitons dissociate thermally. The electric field is also strongly altered by the electronic type of outer wall. The metallic walls act as a dense dielectric shield, blocking a substantial fraction of the electromagnetic field. That manifests in ≤30 times weaker Raman signals of the inner metallic walls compared to those of the outer metallic walls. These results open interesting prospects for dielectric cloaking and active dielectric screening. We believe that in all types of 1D heterostructures, one will find strong dielectric effects altering many-body interactions and electromagnetic fields.

## Supporting Information Available

Experimental methods, detailed Raman intensity calculation, RBM frequency diameter dependence, Maxwell–Garnet mixing scheme, exciton lifetime broadening comparison, and estimation of the Fermi level in the inner metallic walls (PDF)
