# Supplementary material for: Dielectric Screening inside Carbon Nanotubes
Source: Nano Lett. 2024 Jun 24;24(26):8030–7. doi: 10.1021/acs.nanolett.4c01668 (PMC11229072; doi:10.1021/acs.nanolett.4c01668)
Supplement: Supplementary file 1 — nl4c01668_si_001.pdf [file nl4c01668_si_001.pdf]

Supporting Information: Dielectric Screening Inside Carbon Nanotubes

Georgy Gordeev,[*,†,‡] Sören Wasserroth,[†] Han Li,[¶] Ado Jorio,[‖] Benjamin S. Flavel,[¶] and Stephanie Reich [*,†]

†*Department of Physics, Freie Universität Berlin, Arnimallee 14, 14195 Berlin*

‡*Department of Physics and Materials Science, University of Luxembourg, Rue du Brill 41, L-4422 Belvaux, Luxembourg*

¶*Institute of Nanotechnology, Karlsruhe Institute of Technology, Hermann-von-Helmholtz-Platz 1, 76344 Eggenstein-Leopoldshafen, Germany*

§*Department of Mechanical and Materials Engineering, University of Turku, Vesilinnantie 5, 20500 Turku, Finland*

‖*Departamento de Física, Universidade Federal de Minas Gerais, Belo Horizonte, Minas Gerais 30123-970, Brazil*

E-mail: georgy.gordeev@uni.lu; stephanie.reich@physik.fu-berlin.de

Experimental Methods

To study the dielectric screening effects in the inner walls, we need to sort as-grown DWCNTs into fractions according to the electronic character of the inner and outer wall.[1] We sorted the (inner@outer) DWCNTs using a three-step technique into the electronic fractions M@M,M@S, S@M, and M@M, where M indicates metallic and S semiconducting character of the wall. The DWCNTs were filtered first in a gel permeation chromatography column by monitoring the Raman intensity. Second, the DWCNTs were re-suspended in toluene and

chlorobenzene with PFO-BPy polymer for improved outer-wall separation. At the final step pellets were extracted from solutions by 1 hour centrifuging at up to 10^6g and deposited onto silicon substrates.[1] After drying, the samples were used for resonant Raman experiments. The Raman maps from four electronic fraction were used to confirm the purity of the samples in ref. Li et al.[1] The highest purity is found in the S@S,M@M, and M@S samples, whereas in the S@M sample we observed undesirable signal from semiconducting outer walls originating from the E_{33} transition (1.6 nm, 2.3 eV). Further the average diameter of the inner walls was higher compared to the S@S sample with an average of 1 nm.

Transition energies and electromagnetic screening were analyzed with resonant Raman spectroscopy of the radial breathing mode (RBM).[2,3] Two excitation-tunable lasers were used as excitation sources, for the visible excitation range 570-670nm a Radiant dye laser (DCM, R6G) and a Coherent Ti-Sa laser for near infra-red excitation (700-850nm). The laser was focused onto the sample using a 100x microscope objective (N.A. 0.9) with position and focus optically controlled by a camera. The back-scattered light was filtered by a Horiba triple grating t64000 system to remove the Rayleigh light and dispersed by 600 and 900 grooves/mm gratings. A Peltier cooled charge-coupled device was detecting the Raman signals. The spectra of 532 nm laser line were acquired with a Horiba Xplora, single-grating spectrometer equipped with a dichroic mirror. The (n, m) chiralities were identified and RBM shifts were investigated systematically using multi-peak fitting. The concept of laola family facilitated chiral identification, the (n, m) from the same laola group share the parameter $l = 2n + m$. In M@M and M@S samples we found the $l = 2m + n = 24$ laola groups – containing the (n, m) chiralities (9,6), (10,4), (11,2), and (12,0) – plus the l =27 and 30 laola groups. A CaF_2 single crystal reference spectrum was measured for each laser wavelength. The integrated area of the RBM peaks was divided by the area of CaF_2 peak at 320 cm^{-1} in order to account for changes in the sensitivity of optical components in the measurement.[2] This procedure also eliminates the pre-factor E_l^4 in Eq. 4.

Variation of Raman intensity in SWCNTs and in the M@M sample

In this section we explain intensities variation in the single walled carbon nanotubes and how local factor can be implemeted. The population is modelled by a standard Gaussian function:

$$I_{i,o}(d) = Ae^{-\frac{(d-b)^2}{2c^2}}, \tag{1}$$

where A reflects the concentration, b average diameter, and c diameter distribution.

We also need to account for chiral variation of matrix elements.[4] For SWCNTs can use empirical formulas proposed by *Pesce et al.*[5]

$$M_R(d) = \left(M_A + \frac{M_B}{d} + \frac{M_C \cos(3\Theta)}{d^2} \right)^2, \tag{2}$$

where M_i with $(i = A, B, C)$ are constants $(M_A = 1.68eV, M_B = 0.52nm \cdot eV, M_C = nm^2 \cdot eV)$ deduced by correlating transmission electron microscopy (TEM) and resonance Raman scattering,[5] d is the CNT diameter and Θ its chiral angle.[6]

The full profile of Raman matrix elements can be described as

$$M_R(d) = M_R(d) \cdot (f^4_{loc}(\epsilon_{eff}) I_i(d) + I_o(d)), \tag{3}$$

Transition energies of the CNTs can be calculated using following empirical formula[7]

$$E_{ii}(p, d) - \beta_p cos3\theta/d^2 = a\frac{p}{d}\left[1 + b \cdot log\frac{cd}{p}\right], \tag{4}$$

where p = (1,2,3,4,5) for (E^S_{11}, E^S_{22}, E^M_{11}, E^S_{33}, E^S_{44}), a = 1.049 eV·nm, b = 0.456, and c = 0.812 nm^{-1}. The β_p parameter depends on the transition number and CNT type. The CNT is of type 1 when $(2n+m)mod3 = 1$ and of type 2 if $(2n+m)mod3 = 2$. The β_p equals (-0.07,0.05), (0.19,-0.14), (-0.19), (-0.42,0.42), and (0.4,-0.4) for p = 1,2,3,4, and 5 respectively.

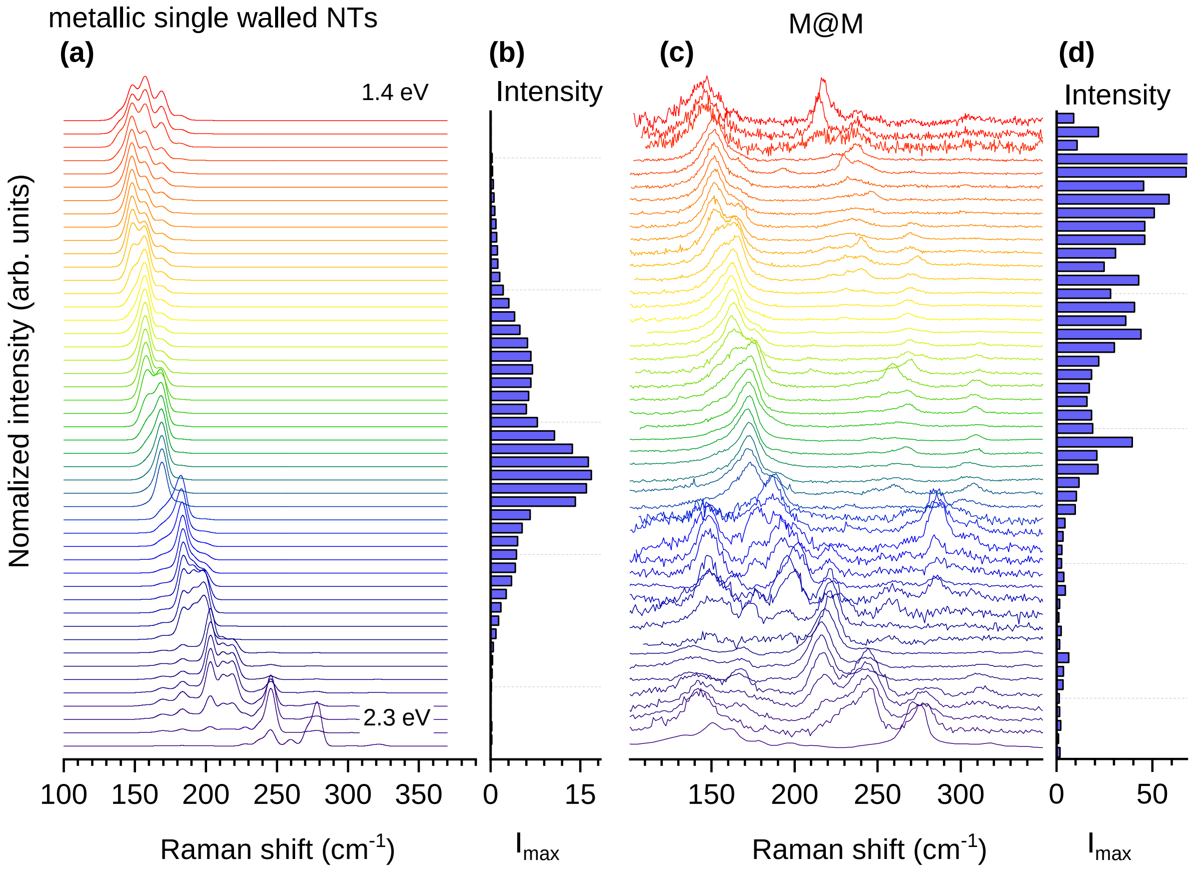

Figure S1: Resonant Raman spectra of nanotubes excited between of 2.3 eV (blue) and 1.55 eV (red), the intermediate colors are mixed in rainbow-like pattern. The panel a) represents the single-walled CNTs, generated for the same populations as M@M walls using eqs. (5),(4), and (2).[5,7] The spectra are normalized and offset for clarity, b) the normalization factors for each laser wavelength. c) Experimental spectra of M@M sample, with d) histograms of the maximum Raman intensity.

In Fig S1a, c we show the waterfall plots for SWCNT and M@M sample, the RBMs belonging to different chiralities move inside and outside the resonance conditions, depending on the laser energy. The SWCNT spectra were obtained using known intensity and transition energies behaviors[5,7] and the M@M are the experimental. The spectra are normalized to one for better visibility and the normalization factors are shown in Figs S1b,d. In the SWCNT sample the rbms originating from inner walls have larger cross sections due to their smaller diameter.[5] Interestingly, the intensities distributions are inverse for the M@M sample, where we find largest intensity for the large diameter walls, red region in Fig S1b. This inversion clearly indicates dielectric effects that reduces the intensities from the inner walls. The M@M an ideal sample, since both inner and outer walls are excited at the same transition M_{11}. In order to quantify the screening effects we need to identify individual (n,m) chiralities from the groups the RBM peaks in Fig S1 as we explain in the main text.

Dependence of the RBM frequency on the CNT diameter

The dependence of the RBM frequency depends on the effective mass of the CNT cylinder, traditionally expressed as:[2]

$$\omega_{RMB}(d) = c_1/d + c_2, \tag{5}$$

where c_1 and c_2 are constants determined from an experiment. Later, a more physical expression was proposed:[8]

$$\omega_{RMB}(d) = 227\sqrt{(\frac{1}{d^2} + \frac{6(1-v^2)}{Eh}\frac{K}{s_0^2})}, \tag{6}$$

where $\frac{6(1-v^2)}{Eh} = 26.3$ meV is intrinsically determined constant and $\frac{K}{s_0^2}$ incorporates interaction forces with the CNTs environment. We combine these two constants into c_2 to simplify the

expression:

$$\omega_{RMB}(d) = 227\sqrt{(\frac{1}{d^2} + c_a)}, \tag{7}$$

where $c_a = \frac{6(1-v^2)}{Eh}\frac{K}{s_0^2}$. Fig S2 shows the fit by Eq. (7). We obtain $c_a^{ms} = 2.85$, $c_a^{mm} = 2.5$, both slightly higher than c_a^{swcnts} 2.2.[8]

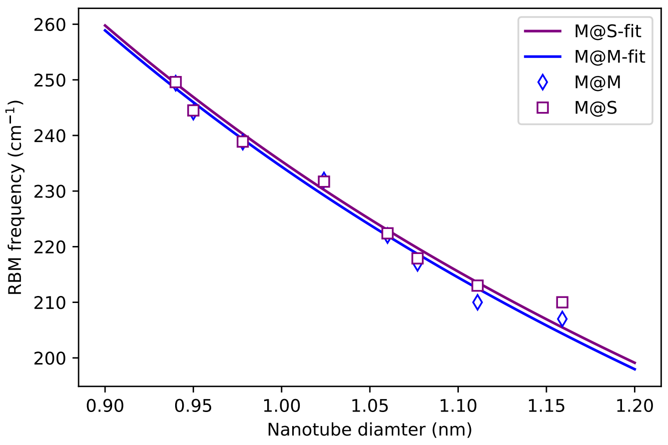

Figure S2: Fit of the experimental RBM frequencies with Eq. (7)

Maxwell-Garnett mixing

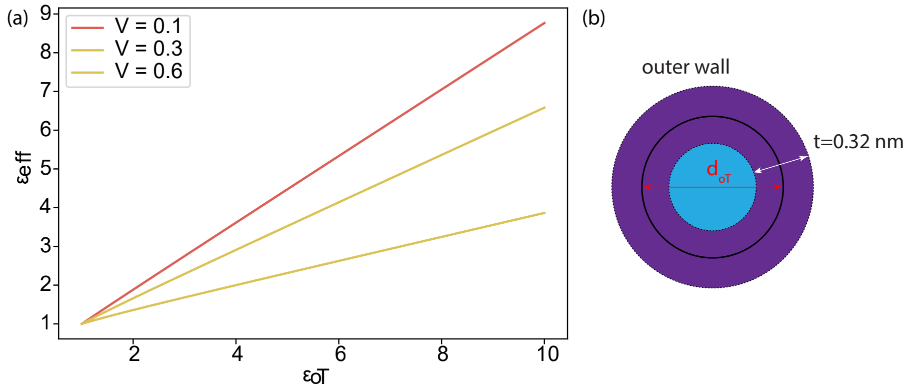

Figure S3: (a) Dependence of ϵ_{eff} on the outer wall dielectric constant ϵ_{oT}, for different volume fractions calculated by Eq. (8) and (b) scheme of volume fraction calculation.

Table S1: Summary of RBM frequencies $\hbar\omega_{M@S}$ and transition energies $E_{11(L)}^{M@X}$, where $X = M$ for metallic inner wall and $X = S$ for semiconducting inner wall. $\Delta E_{11(L)}^{M@X} = E_{11}^{SW} - E_{11}^{DW}$. $\Delta E_{11(L)}^{M@X}$ is $E_{11(L)}^{M@M} - E_{11(L)}^{M@S}$ .The data for SWCNTs is a combination of empirical and calculated values from *Maultzsch et al.*,[2] see Methods. In Eq. (5) $c_2^{M@M} = 19.3nm$, $c_2^{M@S} = 20.2nm$, and $c_1 = 215cm^{-1} \cdot nm$

chirality (n,m)	ω_{SW} cm^{-1}	E_{SW} eV	$\hbar\omega_{M@M}$ cm^{-1}	$E_{11(L)}^{M@M}$ eV	$\hbar\omega_{M@S}$ cm^{-1}	$E_{11(L)}^{M@S}$ eV	$\Delta E^{X@M}$ meV	$\Delta E_{11(L)}^{M@M}$ meV	$\Delta E_{11(L)}^{M@S}$ meV	$\Delta\hbar\omega$ cm^{-1}
l=24										
(9,6)	228	2.25	232.0	2.134	231.7	2.156	22	-116	-94	3.7
(10,4)	238	2.24	238.8	2.132	238.9	2.172	40	-108	-68	0.9
(11,2)	244	2.22	244.2	2.128	244.5	2.163	35	-92	-57	0.5
(12,0)	247	2.21	249.4	2.128	249.6	2.152	25	-82	-58	2.6
l=27										
(10,7)	203	2.04	207	2.050	210	2.070	21	10	30	7
(11,5)	211	2.08	210	2.050	213	2.062	12	-30	-18	2
(12,3)	217	2.07	217	2.037	217.9	2.049	12	-33	-21	0.9
(13,1)	221	2.07	222	2.024	222.4	2.034	10	-46	-36	1.4
l=30										
(13,4)	193.5	1.93	195.9	1.902	197.6	1.899	-2	-28	-31	4.1
(14,2)	196.3	1.92	199.8	1.901	201.9	1.896	-6	-19	-24	5.6
(15,0)	200.4	1.88	204.4	1.904	206.0	1.894	-10	24	14	5.6

We use the following formula of Maxwell-Garnet mixing[9]

$$\epsilon_{\text{eff}} = \epsilon_{oT} + 3V\epsilon_{\text{oT}}\frac{\epsilon_i - \epsilon_{\text{oT}}}{\epsilon_i + 2\epsilon_{\text{oT}} - V(\epsilon_i - \epsilon_{\text{oT}})}. \tag{8}$$

where $V(d) = 1 - 4/(d+t)^2$ is the volume fraction of the empty part of the cylinder. Eq (8)is plotted in Fig S3 for various V parameters.

The volume fraction V refers to the ratio of the outer nanotube empty V_{emp} part to the outer nanotube 'full' volume V_{full}. The scheme is shown in Fig S3, with empty part coloured in blue and filled part coloured in purple. The empty part is $\pi(r_{\text{oT}} - t/2)^2h$, whereas the full part is $\pi(r_{\text{oT}} + t/2)^2h$, with h being the nominal length of the nanotube and r its radius. The overall volume of the unfilled to the total volume is therefore:

$$V_{emp} = \frac{V_{unfilled}}{V_{total}} = \frac{(r_{\text{oT}} - t/2)^2}{(r_{\text{oT}} + t/2)^2} = \frac{(d_{\text{oT}} - t)^2}{(d_{\text{oT}} + t)^2}. \tag{9}$$

Exciton lifetime broadening comparison

In this section we estimate the impact of the SWCNTs impurities using the analysis of the exciton lifetime broadening. After After the first purification steps a sonication is performed that can potentially extract the inner walls, creating the SWCNT impurities.[10] These impurities can contribute to the overall Raman signal. We use the analysis of the exciton broadening to estimate the contribution of such impurities, where in bundled SWCNTs broader profiles are expected.[11]

Fig S4a,d compares the resonant Raman profile of the same (9,4) semiconducting SWCNT(s-SWCNT) chirality suspended and inside a bundle. When suspended the broadening of the Raman profile in eq. (5) γ is only 30 meV.[11] Inside a heterogeneous bundle O'Connell et al.[11] report an increase of $\gamma = 103$ meV. We found similar values both in surfactant wrapped and water suspended s-SWCNTs[12] with $\gamma = 43$ meV and bundles of m-SWCNTs[13] inside the films with $\gamma = 90$ meV, the Raman profiles are shown in Fig S4b,e.

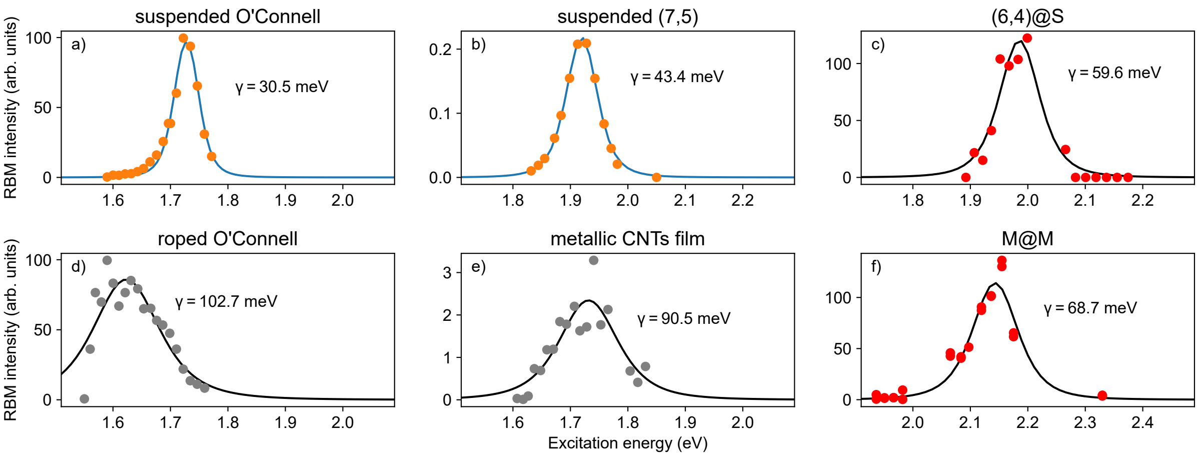

Figure S4: Resonant Raman profiles of a) individual (9,4) SWCNTs, and d) (9,4) s-SWCNTs bundles mixed with outer chiralities, adapted from O'Connell et al.[11]. b) The RR profile of the water suspended (7,5) s-SWCNT[12] and e) m-SWCNT in mixed chirality film.[13] c) RR profile of (6,4)@S with $\omega_{rbm} = 346\,\mathrm{cm}^{-1}$ indicating strong moiré coupling condition.[14] f) RR profile of the (11,2)@M DWCNTs from the M@M sample.

In the M@M and S@S pellets the broadening is smaller compared to the bundels of SWCNTs. We select a profile from the S@S sample with high RBM shift at $346.5\,\mathrm{cm}^{-1}$ that definitely belongs to an inner wall of a DWCNTs.[14] The Raman profile is shown in Fig S4c, after fitting we obtain $\gamma = 60\,\mathrm{meV}$. Next we investigate the (11,2)@M CNT from this work, the broadening of the Raman profile is 69 meV, comparable with the S@S sample and 20-30 meV smaller than expected in heterogeneous bundles of SWCNTs. Therefore, we conclude that the amount of single walled impurities is low, compared to M@M DWCNTs.

Table S2: Fitting parameters of the broadening profiles from eq. (5). The raw data for roped(bundled) s-SWCNTs from,[11] suspended (7,5) from,[12] m-SWCNTs film from[13] and S@S from.[14]

sample	ω_{rbm} (meV)	M (arb. unit)	γ (meV)	E_{ii} (eV)
suspended O'Connell[11]	31.7	0.011	30.5	1.713
roped O'Connell[11]	20.6	-0.013	90.5	1.717
Mfilm[13]	31.7	0.1	102.7	1.608
(7,5)[12]	35.0	0.001	43.4	1.905
(6,4)@S[14]	42.9	0.041	59.6	1.97
(11,2)@M	30.3	0.053	68.7	2.128

Estimation of the doping level in the inner walls

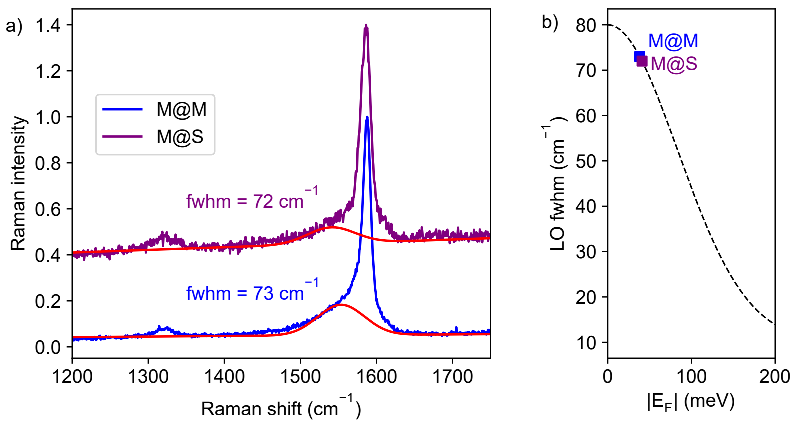

Figure S5: Doping effect in the metallic inner walls. a) Raman spectra of the M@M and M@S samples excited with 1.96 eV laser, red line indicated the LO phonon. b) LO phonon width as function of Fermi level shift abmlitude $|E_F|$ for $d = 1.1$ nm.

In this section we calculate the contribution of the doping to the intensity changes in the metallic inner walls. The G mode in metallic and semi-metallic carbon nanotubes is sensitive to the doping. We used this property of the G mode in several works for estimating the Fermi level in functionalized CNTs[15,16] and in the CNT-gold nanoparticle hybrids.[13] The G mode consist of two types of phonon, longitudinal (LO) and transverse (TO). The LO phonon is coupled to the electronic states and it's shape deviates from Lorenz resonance and resembles Breit-Wigner Fano line shape.[17] The broadening of this mode is at maximum at charge neutrality point and decreases as Fermi level to the positive or to the negative values. The width of the LO phonon at charge the neutrality point decreases with diameter from $145\,\mathrm{cm}^{-1}$ at $d = 0.6$ nm to $50\,\mathrm{cm}^{-1}$ at $d = 2.4$ nm.

We estimate the doping in the inner metallic walls using the analysis of the G mode width. We excite the G mode of M@M and M@S sample with $1.9\,\mathrm{cm}^{-1}$ excitation line. Fig S5a shows the experimental Raman spectra in the high-energy range. The mode $\approx 1553\,\mathrm{cm}^{-1}$ corresponds to the LO phonon (highlighted in red). The phonon full width at half maxima (fwhm) is $72\,\mathrm{cm}^{-1}$ in the M@S sample and $73\,\mathrm{cm}^{-1}$ for the M@M sample. The 1.9 eV laser

resonates with inner walls with an average diameter of ≈ 1.1 nm. This correspond to the LO width of $80\,\mathrm{cm}^{-1}$ at charge neutrality point.[18] The LO fwhm distribution is shown in Fig S5b, the fwhm decreases as Fermi level is moving up or down. The measured width of the LO peak corresponds to the Fermi shift of ≈ 40 meV. This Fermi level is not sufficient to cause a change of Raman intensity more than 80 %.[19,20] Therefore we attribute the main contribution to the dielectric effect.

References

(1) Li, H.; Gordeev, G.; Wasserroth, S.; Chakravadhanula, V. S. K.; Neelakandhan, S. K. C.; Hennrich, F.; Jorio, A.; Reich, S.; Krupke, R.; Flavel, B. S. Inner- and outer-wall sorting of double-walled carbon nanotubes. *Nature Nanotechnology* **2017**, *12*, 1176–1182.

(2) Maultzsch, J.; Telg, H.; Reich, S.; Thomsen, C. Radial breathing mode of single-walled carbon nanotubes: Optical transition energies and chiral-index assignment. *Phys. Rev. B* **2005**, *72*, 205438.

(3) Telg, H.; Maultzsch, J.; Reich, S.; Hennrich, F.; Thomsen, C. Chirality distribution and transition energies of carbon nanotubes. *Physical Review Letters* **2004**, *93*, 177401.

(4) Jiang, J.; Saito, R.; Sato, K.; Park, J. S.; Samsonidze, G. G.; Jorio, A.; Dresselhaus, G.; Dresselhaus, M. S. Exciton-photon, exciton-phonon matrix elements, and resonant Raman intensity of single-wall carbon nanotubes. *Phys. Rev. B* **2007**, *75*, 035405.

(5) Pesce, P. B. C.; Araujo, P. T.; Nikolaev, P.; Doorn, S. K.; Hata, K.; Saito, R.; Dresselhaus, M. S.; Jorio, A. Calibrating the single-wall carbon nanotube resonance Raman intensity by high resolution transmission electron microscopy for a spectroscopy-based diameter distribution determination. *Applied Physics Letters* **2010**, *96*, 051910.

(6) Thomsen, C.; Reich, S. In *Raman Scattering in Carbon Nanotubes.* Light Scattering in Solids IX; Manuel, C., Merlin, R., Eds.; Springer: Berlin Heidelberg, 2007; pp 164–169.

(7) Araujo, P. T.; Jorio, A.; Dresselhaus, M. S.; Sato, K.; Saito, R. Diameter Dependence of the Dielectric Constant for the Excitonic Transition Energy of Single-Wall Carbon Nanotubes. *Physical Review Letters* **2009**, *103*, 146802.

(8) Araujo, P. T.; Maciel, I. O.; Pesce, P. B. C.; Pimenta, M. A.; Doorn, S. K.; Qian, H.; Hartschuh, A.; Steiner, M.; Grigorian, L.; Hata, K.; Jorio, A. Nature of the constant factor in the relation between radial breathing mode frequency and tube diameter for single-wall carbon nanotubes. *Phys. Rev. B* **2008**, *77*, 241403.

(9) Holmström, P.; Thylén, L.; Bratkovsky, A. Dielectric function of quantum dots in the strong confinement regime. *Journal of Applied Physics* **2010**, *107*.

(10) Erkens, M.; Cambré, S.; Flahaut, E.; Fossard, F.; Loiseau, A.; Wenseleers, W. Ultrasonication-induced extraction of inner shells from double-wall carbon nanotubes characterized via in situ spectroscopy after density gradient ultracentrifugation. *Carbon* **2021**, *185*, 113–125.

(11) O'Connell, M. J.; Sivaram, S.; Doorn, S. K. Near-infrared resonance Raman excitation profile studies of single-walled carbon nanotube intertube interactions: A direct comparison of bundled and individually dispersed HiPco nanotubes. *Phys. Rev. B* **2004**, *69*, 023813.

(12) Gordeev, G. Elementary Exciton Mediated Raman Scattering Mechanisms in Pristine and Functionalized Single Walled Carbon Nanotubes. Ph.D. thesis, Freie Universität Berlin, 2019.

(13) Wroblewska, A.; Gordeev, G.; Duzynska, A.; Reich, S.; Zdrojek, M. Doping and plasmonic Raman enhancement in hybrid single walled carbon nanotubes films with embedded gold nanoparticles. *Carbon* **2021**, *179*, 531–540.

(14) Gordeev, G.; Wasserroth, S.; Li, H.; Flavel, B.; Reich, S. Moiré-Induced Vibrational Coupling in Double-Walled Carbon Nanotubes. *Nano Letters* **2021**, *21*, 6732–6739.

(15) Gordeev, G.; Setaro, A.; Glaeske, M.; Jürgensen, S.; Reich, S. Doping in covalently functionalized carbon nanotubes: A Raman scattering study. *Physica Status Solidi (B)* **2016**, *253*, 2461–2467.

(16) Setaro, A.; Adeli, M.; Glaeske, M.; Przyrembel, D.; Bisswanger, T.; Gordeev, G.; Maschietto, F.; Faghani, A.; Paulus, B.; Weinelt, M.; Arenal, R.; Haag, R.; Reich, S. Preserving π-conjugation in covalently functionalized carbon nanotubes for optoelectronic applications. *Nature Communications* **2017**, *8*, 14281.

(17) Brown, S. D.; Jorio, A.; Corio, P.; Dresselhaus, M. S.; Dresselhaus, G.; Saito, R.; Kneipp, K. Origin of the Breit-Wigner-Fano lineshape of the tangential G-band feature of metallic carbon nanotubes. *Phys. Rev. B* **2001**, *63*, 1–8.

(18) Hatting, B.; Heeg, S.; Ataka, K.; Heberle, J.; Hennrich, F.; Kappes, M. M.; Krupke, R.; Reich, S. Fermi energy shift in deposited metallic nanotubes: A Raman scattering study. *Physical Review B - Condensed Matter and Materials Physics* **2013**, *87*, 165442.

(19) Kalbac, M.; Green, A. A.; Hersam, M. C.; Kavan, L. Probing charge transfer between shells of double-walled carbon nanotubes sorted by outer-wall electronic type. *Chemistry - A European Journal* **2011**, *17*, 9806–9815.

(20) Farhat, H.; Sasaki, K.; Kalbac, M.; Hofmann, M.; Saito, R.; Dresselhaus, M. S.; Kong, J. Softening of the Radial Breathing Mode in Metallic Carbon Nanotubes. *Physical Review Letters* **2009**, *102*, 126804.
